# CRISPR technologies for detecting DNA and RNA methylation: Mechanisms, platforms, and translational opportunities

**DOI:** 10.1002/btm2.70174

**Published:** 2026-08-19

**Authors:** Kaixin Chen, Biyao Yang, Rui Sang, Wenjie Chen, Tingxiu Xiang, Fei Deng

**Affiliations:** ^1^ School of Biomedical Engineering University of New South Wales Sydney Australia; ^2^ Australian Centre for NanoMedicine University of New South Wales Sydney Australia; ^3^ The NMPA and State Key Laboratory of Respiratory Disease, School of Pharmaceutical Sciences, Department of Emergency, The Second Affiliated Hospital Guangzhou Medical University Guangzhou People's Republic of China; ^4^ Chongqing Key Laboratory for the Mechanism and Intervention of Cancer Metastasis, Chongqing University Cancer Hospital Chongqing University Chongqing People's Republic of China

**Keywords:** CRISPR diagnostics, DNA methylation, liquid biopsy, point‐of‐care testing, RNA methylation, translational bioengineering

## Abstract

DNA and RNA methylation are key epigenetic and epitranscriptomic modifications involved in gene regulation, genome stability, RNA metabolism, and disease progression. Aberrant methylation patterns in cell‐free DNA and RNA have emerged as valuable biomarkers for cancer detection, disease monitoring, and therapeutic stratification. However, conventional methods such as bisulfite sequencing, methylation‐specific PCR, MeRIP‐seq, SCARLET, and LC–MS/MS often require harsh processing, high sample input, complex instrumentation, or lack site‐specific resolution, limiting their clinical and point‐of‐care applications. CRISPR‐based diagnostics provide a promising alternative by combining programmable nucleic acid recognition with collateral cleavage‐mediated signal amplification. This review summarizes recent CRISPR strategies for detecting DNA and RNA methylation, including chemical conversion‐assisted assays, restriction enzyme‐mediated detection, direct amplification‐free sensing based on methylation‐modulated Cas activity, detection of oxidized cytosine derivatives, reverse transcription‐mediated Cas12 detection of m6A, and structure‐sensitive Cas13 sensing. We highlight how methylation‐dependent sequence conversion, enzyme accessibility, polymerase behavior, and nucleic acid structure can be translated into CRISPR‐readable signals. Finally, we discuss current translational challenges and emerging opportunities in point‐of‐care methylation diagnostics, integrated DNA–RNA profiling, engineered Cas effectors, AI‐guided assay design, and CRISPR‐compatible methylome analysis.

**Translational Impact Statement**

CRISPR‐based methylation detection offers a programmable framework for translating subtle DNA and RNA modifications into rapid, sensitive, and potentially decentralized diagnostic readouts. By integrating modification‐selective chemistry, methylation‐sensitive enzymes, reverse transcription, and structure‐responsive Cas effectors, these platforms could enable minimally invasive analysis of disease‐associated epigenetic biomarkers in cell‐free nucleic acids. This review defines the key engineering, standardization, manufacturing, and clinical validation priorities required to advance CRISPR methylation assays from laboratory proof‐of‐concept systems toward robust and deployable diagnostic technologies.

## 1 INTRODUCTION

Epigenetic regulation provides a multilayered framework for controlling gene expression, cellular identity, and phenotypic outcomes without altering the underlying genomic sequence.^1^ Within this landscape, DNA and RNA methylation constitute two of the most influential layers of epigenetic and epitranscriptomic control. DNA 5‐methylcytosine (5mC) and its oxidized derivatives such as 5‐hydroxymethylcytosine (5hmC), 5‐formylcytosine (5fC), and 5‐carboxylcytosine (5caC) could govern transcriptional repression, genomic imprinting, chromatin accessibility, and cellular memory, while aberrant methylation patterns contribute to tumor suppressor gene silencing, genome instability, and malignant transformation.^2^ In parallel, RNA methylation, especially N^6^‐methyladenosine (m6A), dynamically regulates RNA splicing, stability, translation, and immune recognition.^3^ Additional RNA marks such as 5‐methylcytosine (m5C), 1‐methyladenosine (m1A), 7‐methylguanosine (m7G), and 3‐methylcytosine (m3C) further diversify the regulatory network and are increasingly implicated in cancer progression, immune modulation, neurological disorders, and viral infection.^4^ Together, DNA and RNA methylation form an interconnected epigenetic–epitranscriptomic regulatory system that underpins normal development and disease pathogenesis. Their measurable alterations in tissues and body fluids highlight their potential as biomarkers for early cancer detection, minimal residual disease monitoring, and precision therapeutic stratification.

Despite this biological significance, current analytical approaches for methylation remain limited in sensitivity, scalability, and clinical deployability. Traditional DNA methylation assays including bisulfite sequencing, methylation‐specific PCR, pyrosequencing, methylated DNA immunoprecipitation, and methylation‐sensitive restriction enzymes require harsh chemical treatments, complex workflows, and specialized equipment, making their application to low‐input clinical samples challenging.^5^ RNA methylation detection faces even greater barriers: MeRIP‐seq lacks nucleotide‐level precision, LC–MS/MS offers only global quantification, and SCARLET (Site‐specific Cleavage And Radioactive Labelling followed by Ligation‐assisted Extraction and TLC) is technically demanding and unsuitable for routine or high‐throughput analysis.^6^ These constraints hinder the broad adoption of methylation biomarkers in minimally invasive diagnostics such as cfDNA/cfRNA liquid biopsy and point‐of‐care testing.

CRISPR‐based biosensing has emerged as a transformative solution to these long‐standing limitations. Programmable nucleic acid recognition combined with robust collateral cleavage activities of Cas12 and Cas13 enables rapid, modular, and ultrasensitive detection of specific molecular features.^7^, ^8^ Importantly, mechanistic studies reveal that methylation can modulate CRISPR activation through altered hybridization energetics, changes in R‐loop formation, and methylation‐sensitive reverse transcription. For instance, methylated DNA can induce weaker Cas12a activation than unmethylated DNA,^9^ while m6A in RNA can modulate RT (reverse transcription) read‐through or alter RNA secondary structure in ways that render methylation detectable by Cas13.^10^ When coupled with cascade CRISPR amplification strategies such as Cas13 → Cas12 or Cas13 → Csm6, these systems achieve attomolar‐level sensitivity and enable fine discrimination of methylation states across DNA and RNA substrates.^11^, ^12^

Several review articles have surveyed aspects of methylation biology and detection. Cancer‐focused reviews comprehensively cover DNA methylation pathways and traditional analytical methods such as bisulfite sequencing, MSP, MeDIP, and NGS profiling.^13^ In parallel, CRISPR‐oriented reviews summarize emerging Cas12‐based strategies for locus‐specific DNA methylation detection.^14^ However, these works remain fragmented: existing CRISPR reviews largely overlook RNA methylation and Cas13‐based sensing mechanisms, while cancer epigenetics reviews seldom incorporate CRISPR‐based technologies. More importantly, a unified framework that integrates both DNA and RNA methylation detection is still lacking, despite growing recognition that these modifications jointly influence tumor evolution and immune responses. To address this gap, this review provides a mechanism‐driven synthesis of CRISPR technologies for detecting both DNA and RNA methylation, with a focus on platform designs, signal‐conversion strategies, and their translational potential in next‐generation epigenetic diagnostics.

## 2 BIOLOGICAL, CHEMICAL, AND METHODOLOGICAL FOUNDATIONS OF DNA AND RNA METHYLATION

### 2.1 Enzymatic pathways generating DNA and RNA methylation

#### 2.1.1 DNA methylation pathways

DNA methylation involves the covalent addition of a methyl group to the 5‐position of cytosine to form 5‐methylcytosine (5mC).^15^ This reaction is catalyzed by DNA methyltransferases (DNMTs): DNMT1 primarily maintains existing methylation patterns during DNA replication, whereas DNMT3A and DNMT3B establish de novo methylation marks.^16^ The 10–11 translocation enzymes TET1, TET2, and TET3 sequentially oxidize 5mC to 5‐hydroxymethylcytosine (5hmC), 5‐formylcytosine (5fC), and 5‐carboxylcytosine (5caC) (Table 1). These oxidized derivatives can function as regulatory marks or intermediates in active DNA demethylation through base excision repair.^17^ Among them, 5hmC is relatively abundant in neuronal tissues and stem cells, whereas 5fC and 5caC generally occur at substantially lower levels.^18^ Their distinct abundance and chemical properties provide different opportunities and challenges for selective conversion, enrichment, and detection.

**TABLE 1:** Key features of cytosine modifications relevant to epigenetic regulation.^19^, ^20^

Modification	Enzyme(s) involved	Biological role	Detectability	Diagnostic relevance
5‐methylcytosine (5mC)	DNMT1, DNMT3A/3B	Gene silencing, X‐inactivation, genome stability	High	Strong; biomarker in cancer and other diseases
5‐hydroxymethylcytosine (5hmC)	TET1/2/3	Active demethylation, cell differentiation	Moderate	Emerging marker for tumor subtype and progression
5‐formylcytosine (5fC)	TET enzymes	Intermediate in demethylation	Low	Low; niche studies
5‐carboxylcytosine (5caC)	TET enzymes	Demethylation via BER pathway	Low	Low; mechanistic interest

DNA methylation is distributed non‐uniformly across CpG islands, CpG shores and shelves, enhancers, repetitive elements, and other genomic regions.^21^ Disease‐associated alterations, including hypermethylation of tumor suppressor gene promoters such as *RASSF1A*, *SHOX2*, and *SFRP1*, as well as global hypomethylation of repetitive elements, are well‐established features of multiple cancers.^22^ DNA methylation contributes to transcriptional regulation, chromatin organization, genomic imprinting, X‐chromosome inactivation, and genome stability.^2^ Its relative stability, heritability, and association with pathological changes make it a valuable biomarker for cancer detection, prognosis, and liquid biopsy‐based monitoring.

#### 2.1.2 RNA methylation pathways

RNA methylation encompasses more than 100 chemically distinct nucleoside modifications across multiple RNA species (Table 2). Among these, N^6^‐methyladenosine (m6A) is the most abundant internal modification in eukaryotic mRNA and long non‐coding RNA and is commonly enriched at DRACH motifs. Other prevalent modifications, including 5‐methylcytosine (m5C), 1‐methyladenosine (m1A), and 7‐methylguanosine (m7G), occur in mRNA, tRNA, rRNA, and miRNA and can influence RNA structure, stability, and translation.^24^

**TABLE 2:** Summary of RNA methylation types.^23^

Modification type	Chemical position	RNA types	Biological functions	Enzymes involved
m6A	N6 of adenosine	mRNA, rRNA, tRNA, lncRNA	Regulates splicing, translation, stability; involved in cancer, immunity	Writers: METTL3/METTL14; Erasers: FTO, ALKBH5; Readers: YTHDFs
m5C	C5 of cytosine	mRNA, tRNA, rRNA, ncRNA	Stabilizes RNA; regulates gene expression and embryogenesis	NSun2, NSun6, DNMT2
m1A	N1 of adenosine	tRNA, rRNA, mRNA	Affects RNA secondary structure, translation; linked to cancer	TRMT6/61, ALKBH1/3
m7G	N7 of guanosine	mRNA (5′ cap), tRNA, rRNA	Promotes mRNA stability and translation initiation	METTL1‐WDR4 complex
m3C	N3 of cytosine	tRNA, mRNA	Regulates tRNA function and cell cycle; implicated in tumorigenesis	METTL8, METTL6

These modifications are regulated by modification‐specific writers, erasers, and readers. The METTL3‐METTL14 complex catalyzes m6A deposition with the support of adaptor proteins such as WTAP, VIRMA, and RBM15,^25^ whereas m5C is primarily installed by NSUN family enzymes,^26^ and m1A by methyltransferases including TRMT6/61A and TRMT61B.^27^ Some RNA modifications are reversible^28^; for example, FTO and ALKBH5 mediate the demethylation of m6A, while ALKBH1 acts on m1A and other substrates.^29^ Modified nucleotides are recognized by reader proteins, including YTH domain proteins,^30^ IGF2BP and hnRNP family members,^31^ as well as proposed m5C readers such as ALYREF,^32^ thereby influencing RNA processing, stability, and translation.

Through these regulatory mechanisms, RNA methylation participates in diverse physiological and pathological processes, including development, immune responses, tumorigenesis, and viral replication.^33^ Compared with DNA methylation, RNA methylation is generally more dynamic and occurs within structurally heterogeneous and relatively unstable molecules. Differences among RNA modifications in abundance, base‐pairing properties, structural effects, and enzymatic recognition create distinct analytical challenges and may necessitate modification‐selective conversion, enrichment, or indirect readout before CRISPR‐based detection.

### 2.2 Chemical and structural effects of DNA and RNA methylation

#### 2.2.1 Chemical effects

Chemical modifications alter the physicochemical properties of nucleic acids, including hydrogen bonding, base stacking, duplex stability, and polymerase behavior. In DNA, 5‐methylcytosine (5mC) introduces a hydrophobic methyl group into the major groove and can enhance C‐G duplex stability primarily through improved base stacking. Its oxidized derivatives, 5hmC, 5fC, and 5caC, contain progressively more polar functional groups and can produce distinct effects on base stacking and duplex stability.^34^

RNA modifications can have more pronounced effects on base pairing because RNA frequently adopts complex structures involving canonical and non‐canonical interactions. N^6^‐methyladenosine (m6A) can destabilize A‐U pairing by imposing a conformational penalty on duplex formation, thereby altering local thermodynamic stability.^35^ In contrast, methylation at the N1 position of adenosine produces positively charged m1A under physiological conditions, directly disrupting canonical Watson‐Crick base pairing.^36^

These modifications can also affect polymerase activity. DNA polymerases generally tolerate 5mC, whereas 5fC and 5caC may reduce extension efficiency or alter enzyme processivity.^37^ Certain RNA modifications can generate reverse‐transcription signatures, including changes in readthrough efficiency, pausing, misincorporation, or premature termination. These effects are particularly pronounced for base‐pair‐disrupting modifications such as m1A, whereas m6A‐dependent signatures may require appropriately selected reverse transcriptases or reaction conditions.^38^ Collectively, modification‐dependent effects on duplex stability and polymerase behavior provide measurable chemical and enzymatic signatures for downstream methylation analysis.

#### 2.2.2 Structural effects

Beyond their effects on hydrogen bonding and base stacking, nucleic acid modifications can alter local conformation, flexibility, and target accessibility. In DNA, 5mC can increase local rigidity and reduce helical flexibility through enhanced base stacking.^39^ It may also subtly alter B‐DNA parameters, including groove width, twist, and roll.^40^ By contrast, TET‐oxidized derivatives such as 5hmC introduce more polar functional groups and can produce distinct effects on duplex conformation and rigidity.^41^ Although these structural changes are generally modest, they may influence probe hybridization and enzyme accessibility.

RNA modifications can exert more pronounced structural effects because RNA adopts diverse secondary and tertiary structures. m6A can shift local base‐pairing equilibria and destabilize specific duplex regions, producing conformational changes commonly described as “m6A switches”.^42^ These changes can expose or occlude nearby sequences and thereby alter their accessibility to RNA‐binding proteins, complementary probes, reverse transcriptase, or CRISPR effectors.^43^ Other modifications, including m1A and m5C, may also reshape local RNA folding by disrupting preferred base‐pairing geometries or altering steric and stacking interactions. Consequently, modification‐dependent differences in nucleic acid conformation and target accessibility provide a potential basis for structure‐sensitive detection.

### 2.3 Existing methylation detection methods: Principles, strengths, and limitations

#### 2.3.1 Conventional DNA methylation detection technologies

A wide range of analytical strategies has been developed to interrogate DNA methylation, each exploiting different biochemical or chemical principles (Table 3). The most widely used approach is bisulfite conversion followed by sequencing or PCR‐based analysis, long considered the gold standard for mapping 5mC at single‐base resolution. Sodium bisulfite selectively deaminates unmethylated cytosine to uracil while leaving 5mC intact, enabling methylation‐dependent sequence discrimination after PCR amplification.^47^ Whole‐genome bisulfite sequencing (WGBS) and reduced representation bisulfite sequencing (RRBS) provide genome‐wide and CpG‐enriched methylome maps, respectively, whereas targeted bisulfite PCR or pyrosequencing assays offer cost‐effective locus‐specific quantification.^48^ Despite their precision, bisulfite‐based methods suffer significant drawbacks: DNA fragmentation, loss of sequence complexity, harsh reaction conditions, and technical challenges in analyzing low‐input or degraded clinical samples such as circulating cell‐free DNA (cfDNA).^49^

**TABLE 3:** Summary of conventional DNA methylation detection technologies.^44^, ^45^, ^46^

Method	Principle	Advantages	Limitations	Clinical relevance
Bisulfite sequencing	Chemical conversion of unmethylated cytosines to uracil	Single‐base resolution, widely validated	DNA degradation, time‐consuming, low compatibility with cfDNA	High (gold standard)
Methylation‐specific PCR (MSP/qMSP)	PCR amplification of bisulfite‐treated DNA using methylation‐specific primers	Locus‐specific, relatively simple	Incomplete conversion, false positives, limited scope	Moderate–high
MSRE‐PCR	Digestion of DNA with methylation‐sensitive restriction enzymes followed by PCR	No chemical treatment, fast and low cost	Restricted to enzyme recognition sites, low resolution	Moderate
MeDIP/MBD‐Seq	Enrichment of methylated DNA using antibodies or methyl‐binding domains	Genome‐wide profiling, no bisulfite required	Low resolution, CpG bias, high input required	Research use, low clinical adoption

Complementary to chemical conversion, methylation‐specific PCR (MSP) and its quantitative variant (qMSP) rely on primer sets designed to selectively amplify either methylated or unmethylated bisulfite‐converted sequences.^50^ MSP‐based assays are highly sensitive and well suited for screening clinically relevant targets such as *RASSF1A*, *SEPT9*, *SHOX2*, and *WIF1*,^51^ which are commonly hypermethylated in multiple cancers, including lung, colorectal, and nasopharyngeal carcinoma. However, these methods remain locus‐limited, require high‐quality bisulfite‐treated DNA, and cannot provide genome‐wide context.

Another major class of assays is based on methylation‐sensitive restriction enzymes (MSREs), which cleave unmethylated sequences adjacent to specific recognition sites. Methylated DNA remains intact and can be selectively amplified or detected, providing a straightforward yes/no readout of methylation status at enzyme‐accessible loci.^52^ MSRE assays are simple and bisulfite‐free, but they are restricted to CpG sites coinciding with enzyme recognition motifs, limiting their genomic coverage and flexibility.

Antibody or protein‐based enrichment approaches, such as methylated DNA immunoprecipitation (MeDIP) and methyl‐CpG binding domain (MBD)‐based capture, isolate methylated fragments for downstream sequencing or quantification. These enrichment methods enable broader interrogation of methylation landscapes without chemical treatment, but they lack single‐nucleotide resolution, exhibit antibody affinity biases, and require substantial DNA input.^53^

High‐throughput next‐generation sequencing (NGS) approaches, including whole‐genome bisulfite sequencing (WGBS), reduced‐representation bisulfite sequencing (RRBS), oxidative bisulfite sequencing (oxBS‐seq), and TET‐assisted bisulfite sequencing (TAB‐seq), enable base‐resolution discrimination of 5mC from its oxidized derivatives, such as 5hmC. This capability has been widely exploited to interrogate active DNA demethylation pathways and the dynamic regulation of cytosine modifications.^54^ Nonetheless, these methods demand expensive instrumentation, complex library preparation, extensive bioinformatic pipelines, and large DNA quantities, making them impractical for rapid diagnostics or point‐of‐care applications.

Overall limitations across these methods include bisulfite‐induced DNA degradation, locus constraints inherent to MSP and MSRE assays, strong reliance on specialized sequencing infrastructure, and limited compatibility with low‐abundance cfDNA or field‐deployable testing. These challenges highlight the need for methylation detection strategies that can operate with minimal sample input, high specificity, and simplified workflows.

#### 2.3.2 Conventional RNA methylation detection technologies

Detection of RNA methylation presents distinct analytical challenges owing to the chemical diversity, structural dynamism, and rapid turnover of RNA modifications (Table 4). The most widely adopted transcriptome‐wide mapping method is methylated RNA immunoprecipitation sequencing (MeRIP‐seq, or m6A‐seq), which enriches methylated RNA fragments using anti‐m6A antibodies prior to sequencing. This technique has been foundational in establishing global m6A landscapes across tissues and disease states; however, it suffers from inherently low spatial resolution, typically identifying regions of ~100 nucleotides rather than pinpointing individual modified bases.^57^ The approach is further limited by antibody affinity biases, substantial background noise, and poor quantitative reproducibility.^58^

**TABLE 4:** Summary of conventional RNA methylation detection technologies.^55^, ^56^

Method	Principle	Advantages	Limitations	Clinical relevance
MeRIP‐seq/m6A‐seq (antibody‐based enrichment sequencing)	Antibody enrichment + sequencing	Transcriptome‐wide profiling	Low resolution; antibody bias/noise; weak quantitation	Research use, low clinical adoption
LC–MS/MS (mass spectrometry)	Nucleoside hydrolysis + MS quantification	High chemical specificity; absolute quantitation; multi‐mark	No site information; high instrument/input demands	Moderate clinical adoption
SCARLET (site‐specific validation)	Site‐specific cleavage + labeling + TLC	Most definitive site validation	Very low throughput; labor‐intensive; technically demanding	Research use, low clinical adoption
miCLIP/m6A‐CLIP (crosslinking‐based CLIP‐seq)	UV crosslinking + sequencing (mutation/truncation mapping)	High‐resolution mapping	Complex workflow; high input; artifacts/reproducibility issues	Research use, low clinical adoption

Mass‐spectrometry based detection, particularly LC–MS/MS, offers highly accurate quantification of global nucleoside modification levels, enabling absolute measurement of m6A, m5C, m1A, and other marks after RNA hydrolysis. While this method provides chemical specificity unmatched by antibody‐based assays, it cannot determine the sequence context or precise location of modifications. Its reliance on specialized instrumentation, extensive sample preparation, and relatively large RNA input restricts its suitability for locus‐specific analysis or low‐abundance clinical samples such as circulating RNA.^59^

Single‐nucleotide resolution can be achieved using SCARLET, a multi‐step method involving site‐specific cleavage, radioactive labeling, ligation‐mediated extraction, and thin‐layer chromatography. SCARLET delivers unambiguous positional validation of m6A sites but is extremely labor‐intensive, low throughput, and technically demanding, rendering it impractical for large‐scale studies or clinical translation. Crosslinking‐based approaches such as miCLIP and related m6A‐CLIP techniques achieve high positional precision by exploiting UV‐induced crosslinking events that generate characteristic mutations or truncations during reverse transcription. Although powerful, these methods require complex library preparation workflows, large RNA input quantities, and sophisticated bioinformatic pipelines, and often exhibit sequence‐ and structure‐dependent artifacts that compromise reproducibility.^60^

Overall, current RNA methylation detection technologies remain limited by insufficient resolution, high noise, labor‐intensive procedures, and an inability to reliably quantify modification levels at single‐nucleotide resolution. These constraints are particularly restrictive in clinical contexts, where RNA samples are often scarce, degraded, or highly heterogeneous. The methodological gaps illustrate a clear unmet need for rapid, sensitive, and site‐specific RNA methylation detection strategies that require minimal sample input and support translational applications—a need that has catalyzed the development of CRISPR‐based RNA methylation sensing approaches discussed in later sections.

### 2.4 Why DNA and RNA methylation require distinct detection logics

CRISPR effectors generally recognize nucleic acid sequence and structure rather than methylation status itself. DNA methylation must therefore be converted into a difference in target sequence, abundance, or enzymatic accessibility. Chemical conversion can generate distinct sequences from methylated and unmethylated DNA, whereas methylation‐sensitive restriction enzymes can selectively alter target availability.^61^ The relative stability of DNA methylation also facilitates such preprocessing and subsequent sequence‐specific analysis.^62^ These differences can then be recognized by CRISPR effectors through guide‐directed target binding or collateral‐cleavage‐based signal generation.

RNA methylation requires different signal‐conversion strategies because RNA modifications may affect reverse transcription, local conformation, or target accessibility without changing the primary sequence.^62^ Modification‐dependent RT pausing, termination, misincorporation, or readthrough can be converted into differences in cDNA sequence or abundance, whereas structural changes may expose or occlude CRISPR target sites. Modification‐selective chemical reactions or enrichment provide additional routes when intrinsic RT or structural signatures are insufficient. The dynamic and frequently sub‐stoichiometric nature of RNA modifications,^63^ together with the structural heterogeneity of RNA substrates,^63^ further necessitates careful control of sample handling and assay conditions. Thus, DNA methylation assays commonly rely on chemical conversion or enzyme‐mediated discrimination, whereas RNA methylation assays more often exploit RT‐derived signatures, structural accessibility, or modification‐selective preprocessing to generate CRISPR‐readable outputs.

## 3 CRISPR‐BASED PLATFORMS FOR DNA AND RNA METHYLATION DETECTION

### 3.1 CRISPR platforms for DNA methylation detection

**FIGURE 1:**
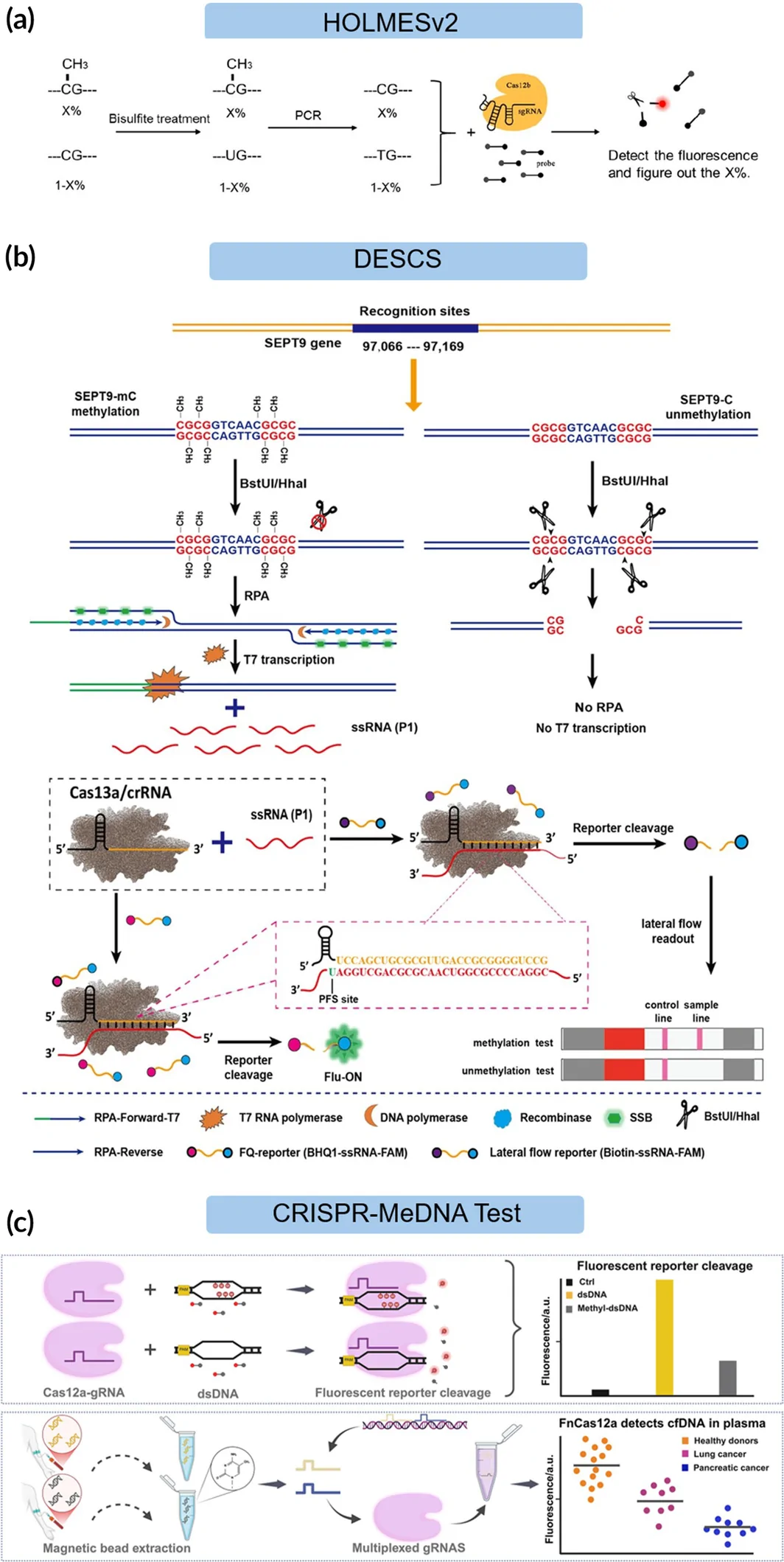
Summary of CRISPR‐based platforms for DNA methylation detection. (a) Schematic illustration of HOLMESv2 workflow (reproduced from Ref. [64]); (b) schematic illustration of the DESCS strategy for the detection of DNA methylation (reproduced from Ref. [65]); (c) schematic illustration of the CRISPR‐MeDNA detection workflow based on Cas12a inhibition by 5mC‐modified cfDNA (reproduced from Ref. [66]).

As conventional CRISPR‐Cas nucleases cannot intrinsically distinguish 5‐methylcytosine (5mC) from cytosine, most CRISPR‐based methylation assays have to first encode methylation status into a sequence‐ or activity‐readable signal that the Cas effector can subsequently transduce through programmable recognition and *trans*‐cleavage. Three mechanistically distinct paradigms have emerged to perform this encoding step, each represented by a landmark platform shown in Figure 1: chemical conversion, in which bisulfite or bisulfite‐free deamination rewrites unmethylated cytosines into thymidines so that 5mC is read out as an SNP‐like base difference (HOLMESv2, Figure 1a)^64^; restriction enzyme‐assisted partitioning, in which methylation‐sensitive endonucleases triage methylated and unmethylated templates by selective cleavage prior to amplification and Cas‐mediated readout (DESCS, Figure 1b)^65^; and direct methylation‐responsive detection, in which 5mC modifications compress Cas‐effector *trans*‐cleavage activity, leading to lower fluorescence signals (CRISPR‐MeDNA Test, Figure 1c).^66^

Together the three paradigms trace a coherent trajectory of diminishing pre‐processing burden, from multi‐step chemical workflows, through one‐step enzymatic preselection coupled to isothermal amplification, to amplification‐ and conversion‐free single‐tube assays compatible with cfDNA‐based liquid biopsy. The representative CRISPR‐based DNA methylation detection platforms reported to date, together with their target loci, assay strategies, sample types, and analytical sensitivities, are further summarized in Table 5.

**TABLE 5:** Summary of existing CRISPR method for DNA methylation detection.

CRISPR	Target	Methods	Sample	LOD	References
Cas12a	Septin9 gene	GlaI‐DC‐SDA‐CRISPR/Cas12a	Human serum	1.28 × 10^−13^ M	^9^
Cas12a	CCDC140 gene	CRISPR‐MeDNA test (amplification‐free)	Human plasma	100 fM	^66^
Cas12a	COL1A2 gene	TET2 oxidizes 5mC to 5caC, followed by Cas12a	Synthetic DNA	‐	^67^
Cas12a	RASSF1A gene	HhaI + PAM‐free RPA + Cas12a	Human serum	0.98 fM	^68^
Cas12a	Genomic DNA	Magnetic beads enrich 5hmC‐DNA, followed by RCA + Cas12a	Cell lines	11 fM	^69^
Cas12a	ACP, CLE, DAB, HOX genes	Bisulfite + PCR + Cas12a	Human serum	–	^70^
Cas12a	Septin9 gene	AciI + Cas12a	CRC tissue and blood	0.4414 nM	^71^
Cas12a	Septin9 gene	GlaI + SDA + Cas12a	CRC tissue and blood	8.19 fM	^71^
Cas12a	Septin9 gene	AciI + Cas12a + DNAzyme	Blood	1.74 nM	^72^
Cas12a	Septin9 gene	AciI + Cas12a + dual‐fluorescence functional nucleic acid reporter	Blood	152 pM	^73^
Cas12a	FEZF2 gene	BstUI + RPA + Cas12a	Human plasma	1 × 10^−8^ ng/μL	^74^
Cas12b	COL1A2 gene	Bisulfite‐PCR‐Cas12b	Cell lines	10^−8^ nM	^64^
Cas13a	SEPT9 gene	Dual MSRE (BstUI+HhaI) + RPA‐Cas13a	Human serum	86.4 aM	^65^

#### 3.1.1 Chemical conversion‐assisted CRISPR methylation detection of 5mC

Chemical conversion strategies represent one of the most powerful approaches for enabling CRISPR to discriminate methylated from unmethylated cytosines. Because CRISPR nucleases cannot directly sense 5mC, these methods create sequence alterations that convert methylation status into nucleotide changes recognizable by Cas proteins. Two principal strategies dominate the field: bisulfite conversion, which chemically deaminates unmethylated cytosines, and bisulfite‐free enzymatic deamination, which reproduces similar C‐to‐U conversions through biologically relevant demethylation pathways.

Bisulfite conversion remains the foundational method for methylation analysis, and its integration with CRISPR enables highly specific and quantitative readouts. Sodium bisulfite selectively converts unmethylated cytosines to uracil while preserving 5mC, generating predictable sequence differences after PCR amplification. These conversion‐induced C → T (or complementary‐strand G → A) sequence changes effectively transform methylation states into SNP‐like targets that can be distinguished by CRISPR nucleases. For example, in the CAM system reported in *Analyst* (2022), bisulfite‐treated DNA undergoes rolling‐circle amplification (RCA) followed by nickase‐mediated signal amplification and Cas12a activation, collectively enabling multi‐layered amplification and discrimination of low‐level methylation.^75^ Similarly, the HOLMESv2 platform employs Cas12b to distinguish bisulfite‐induced sequence variants with high specificity, leveraging the narrower temperature window and higher mismatch sensitivity of Cas12b to quantitatively resolve methylation levels (Figure 1a).^64^ These bisulfite‐driven CRISPR assays benefit from excellent single‐base precision and compatibility with genome‐scale or locus‐specific workflows but retain the drawbacks of bisulfite chemistry—namely DNA fragmentation, sequence loss, and limited suitability for low‐input or clinical samples.

To overcome the destructive nature of bisulfite treatment, recent efforts have focused on bisulfite‐free enzymatic deamination, which mimics natural demethylation pathways. Methods such as meHOLMES employ TET oxidation to convert 5mC to 5hmC, 5fC, or 5caC, followed by APOBEC3A‐mediated deamination, which selectively converts unmethylated cytosines to uracil but leaves modified cytosines intact. This biochemical approach preserves DNA integrity, reduces workflow harshness, and produces clean sequence contexts for CRISPR readout. After PCR amplification, Cas12a can readily differentiate original methylated cytosines from converted uracils, generating a robust *trans*‐cleavage signal with SNP‐level discrimination. Notably, meHOLMES demonstrates improved detection sensitivity compared with bisulfite‐based methods and enables methylation quantification with reduced sample degradation, offering a pathway toward clinically compatible and high‐throughput analysis without reliance on chemical denaturation.^67^

Together, bisulfite‐based and enzymatic conversion‐based CRISPR methods illustrate how methylation‐dependent sequence engineering enables reliable CRISPR readout of DNA methylation. While bisulfite conversion offers maximal chemical separation of methylated and unmethylated cytosines, enzymatic deamination provides a gentler, DNA‐preserving alternative that more effectively supports clinical and low‐input applications. These complementary approaches form a major pillar of methylation‐responsive CRISPR sensing and continue to evolve alongside improvements in amplification chemistries, enzyme specificity, and workflow integration.

#### 3.1.2 Restriction enzyme mediated CRISPR methylation detection of 5mC

Restriction enzyme assisted CRISPR detection is widely adopted early approaches for translating DNA methylation states into CRISPR detectable molecular signatures. These methods exploit the methylation sensitivity of certain restriction endonucleases, enabling selective digestion of either methylated or unmethylated CpG sites.^65^ Two major subclasses have emerged within this strategy: methylation‐sensitive restriction enzymes (MSREs) that selectively cleave unmethylated CpG sites and methylation‐dependent endonucleases such as GlaI that specifically recognize and digest methylated DNA.^14^ Following enzymatic treatment, the digestion pattern determines the availability of amplifiable DNA templates, which are subsequently recognized by Cas12, whose collateral cleavage provides a rapid and sensitive readout.

In the first subclass, MSRE‐based workflows exploit enzymes such as HpaII, HhaI, or BstUI, which are blocked by 5mC at their recognition motifs. As a result, unmethylated DNA is cleaved into short fragments that cannot serve as efficient templates for downstream amplification, whereas methylated DNA remains intact and can be enriched through RPA, PCR, or LAMP prior to Cas12 activation. The differential preservation of amplifiable templates generates a strong methylation‐dependent Cas12 fluorescence output. This principle underlies several MSRE‐CRISPR systems, including a PAM‐independent RPA‐Cas12a assay reported in 2022, which addresses a key practical limitation: many CpG loci lack suitable PAM sites for Cas12 recognition.^68^ By introducing a λ‐exonuclease digestion step that generates single‐stranded DNA intermediates capable of activating Cas12a independent of PAM, this strategy enables flexible detection of diverse CpG loci while maintaining high sensitivity and compatibility with lateral flow readout.^68^ Similar MSRE‐CRISPR architectures have also been applied to cfDNA methylation biomarkers in cancer, where selective preservation of methylated fragments enables quantitative analysis of tumor‐associated hypermethylation patterns in fragmented DNA samples.^76^

A second subclass uses methylation‐dependent nucleases, most prominently GlaI, which selectively cleaves DNA only when CpG sites are methylated. In contrast to MSREs, where unmethylated DNA is preferentially digested, GlaI reverses the logic of signal generation by producing cleavage products exclusively from methylated templates. These fragments can be coupled with isothermal amplification strategies, such as strand displacement amplification (SDA) or rolling circle amplification (RCA), to generate abundant amplicons that subsequently trigger Cas12 *trans*‐cleavage.^77^ For example, the GlaI‐DC‐SDA‐Cas12a platform reported in 2022 integrates methylation‐dependent digestion with a dual‐catalytic amplification cascade, substantially enhancing detection sensitivity and enabling discrimination of low‐abundance methylation targets. This architecture is particularly suited for applications in oncology, where methylation density and fragment abundance vary widely across cfDNA samples.^9^ Importantly, methylation‐dependent nucleases eliminate the need for bisulfite conversion and avoid the sequence constraints associated with MSRE motifs, thereby expanding the range of CpG loci accessible to CRISPR‐based assays.

Together, MSRE‐driven and methylation‐dependent nuclease‐driven strategies demonstrate how selective enzymatic digestion can convert subtle methylation differences into robust, amplifiable molecular signals readily interpreted by CRISPR systems. These enzyme‐assisted approaches are rapid, bisulfite‐free, and compatible with isothermal amplification, making them promising candidates for point‐of‐care methylation diagnostics. However, their applicability remains dependent on the availability, specificity, and sequence constraints of the corresponding restriction enzymes, motivating the development of complementary chemical‐conversion and probe‐engineered CRISPR platforms discussed in subsequent sections.

#### 3.1.3 Direct methylation‐responsive CRISPR detection of 5mC

Unlike conversion‐ or restriction enzyme‐assisted strategies, direct methylation‐responsive CRISPR detection exploits the intrinsic effect of 5mC on Cas effector activation. This emerging approach eliminates the need to first convert methylation status into a sequence difference or selectively digest one methylation state, thereby substantially simplifying the analytical workflow. Instead, methylated and unmethylated targets are distinguished through differences in CRISPR target recognition, R‐loop formation, conformational activation, or collateral cleavage activity.

A recent study demonstrated that site‐specific 5mC modification can directly suppress the *trans*‐cleavage activity of Cas12a. In the CRISPR‐MeDNA Test, methylated target DNA produces substantially weaker Cas12a‐mediated reporter cleavage than the corresponding unmethylated sequence, enabling methylation status to be inferred directly from fluorescence intensity.^66^ Importantly, the magnitude of inhibition is strongly dependent on the position of the methylated cytosine within the crRNA‐target duplex, with methylation at specific PAM‐distal positions producing pronounced suppression of Cas12a activity. Mechanistic analyses further suggested that 5mC alters the conformational dynamics of the Cas12a‐target complex and interferes with productive activation of the nuclease.

This direct activity‐based sensing principle has been extended to amplification‐free analysis of cfDNA. By using multiple gRNAs targeting methylation‐rich regions within the CCDC140 promoter, methylation‐dependent inhibitory effects can be accumulated across several CpG sites, substantially improving analytical discrimination and enabling differentiation of plasma cfDNA from healthy individuals and cancer patients. Compared with conventional CRISPR methylation assays, which generally require bisulfite conversion, restriction digestion, or nucleic acid amplification, this strategy offers a markedly simplified workflow and preserves the native methylation state of the target DNA.

Direct methylation‐responsive CRISPR sensing therefore represents a distinct and potentially transformative paradigm for epigenetic diagnostics. However, its performance remains strongly influenced by methylation‐site position, crRNA sequence, target context, and the magnitude of methylation‐induced Cas inhibition. Further systematic mapping of methylation‐sensitive positions and engineering of Cas effectors with enhanced responsiveness to modified nucleotides will be required to expand this concept from selected loci to broadly programmable methylation detection.

#### 3.1.4 CRISPR detection of 5hmC and other oxidized cytosine derivatives

The TET‐mediated oxidation products 5hmC, 5fC, and 5caC generally preserve Watson‐Crick base pairing and therefore cannot be readily distinguished from unmodified cytosine through conventional CRISPR sequence recognition.^14^ Their detection consequently requires modification‐selective preprocessing to convert the chemical identity of the modified base into differences in target abundance, sequence, or accessibility that can be recognized by CRISPR effectors.

Most reported CRISPR‐based strategies have focused on 5hmC. In the CAICas12a system, 5hmC‐containing DNA is selectively labeled with a photocleavable DBCO‐biotin reagent following modification‐specific functionalization, captured using streptavidin‐coated magnetic beads, and subsequently released for Cas12a‐based analysis.^69^ This enrichment increases the relative abundance of 5hmC‐containing fragments and reduces interference from unmodified DNA. Isothermal amplification methods, such as RCA or SDA, can further convert the enriched targets into abundant Cas12a‐recognizable products, which activate collateral cleavage for sensitive signal generation.^78^ Although analogous chemical conversion or enrichment principles may potentially be adapted to 5fC and 5caC, dedicated CRISPR‐based methods for these less abundant derivatives remain limited. Therefore, current CRISPR detection of oxidized cytosine derivatives remains predominantly centered on 5hmC.

### 3.2 CRISPR platforms for RNA methylation detection

**FIGURE 2:**
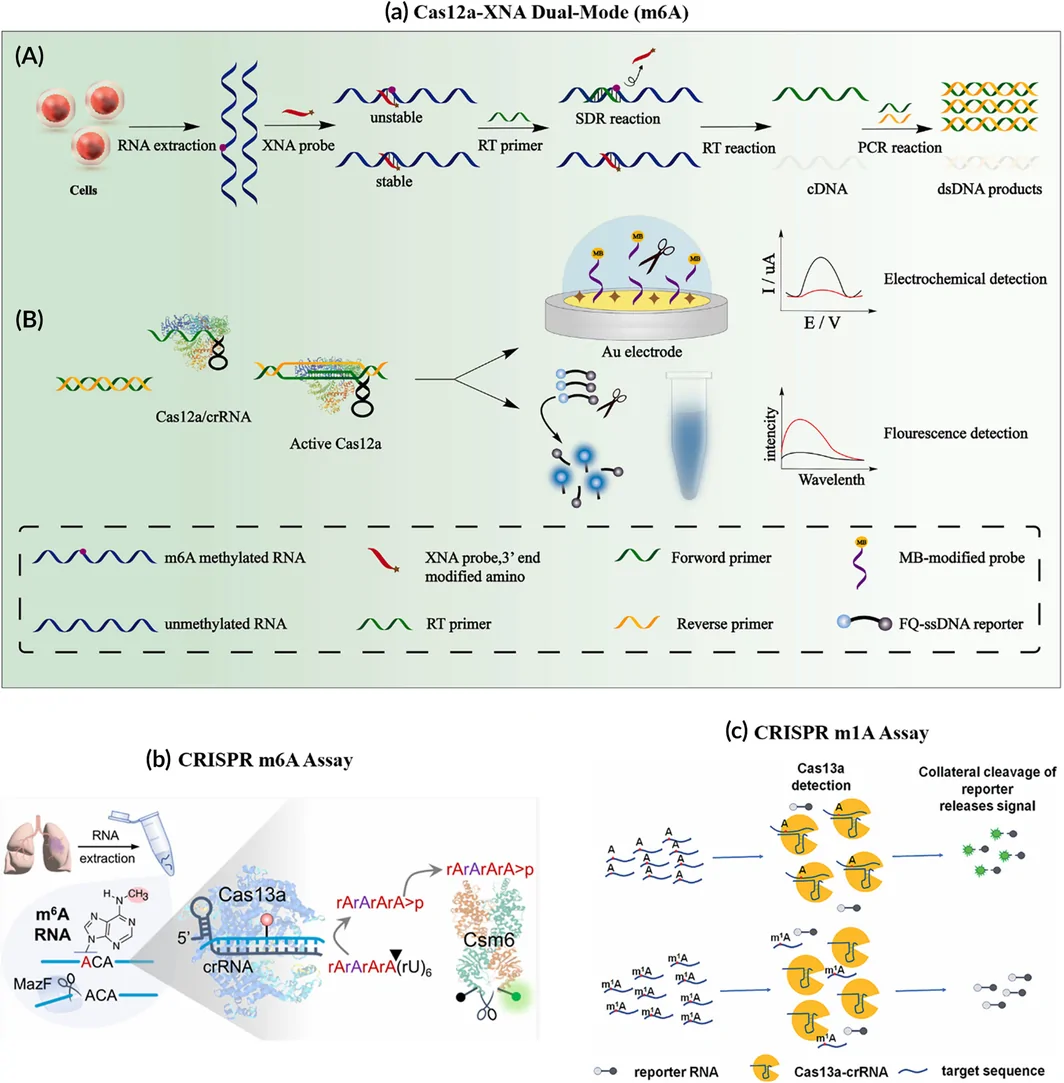
Summary of CRISPR‐based RNA methylation detection systems. (a) Schematic illustration of Cas12a‐XNA dual‐mode workflow for site‐specific m6A RNA detection (reproduced from Ref. [79]). (b) Schematic illustration of the CRISPRm6A assay using MazF‐based m6A recognition coupled with Cas13a‐Csm6 tandem cleavage for site‐specific RNA m6A detection (reproduced from Ref. [80]). (c) Schematic illustration of m1A detection via the CRISPR‐Cas13a system, based on m1A‐induced mismatch suppressing collateral cleavage activity (reproduced from Ref. [81]).

Compared with DNA methylation detection, CRISPR‐based RNA methylation sensing relies on more diverse signal‐conversion mechanisms, including modification‐sensitive reverse transcription, methylation‐dependent enzymatic cleavage, and direct recognition of modification‐induced mismatch or structural effects. Representative CRISPR strategies for site‐specific detection and analysis of RNA methylation are summarized in Figure 2.

#### 3.2.1 Reverse transcription‐mediated Cas12a detection of m6A

RT provides a powerful biochemical bridge for converting RNA methylation marks into detectable sequence or structural signatures compatible with CRISPR‐based readouts, which typically do not alter primary nucleotide identity. RNA modifications, including m6A, m5C, and m1A, disrupt canonical base pairing, alter local RNA structure, or impose steric constraints that modulate polymerase kinetics. These effects manifest during RT as pausing, truncation, misincorporation, or altered extension efficiency, creating modification‐dependent cDNA products that can be selectively amplified and detected using Cas12 nucleases.^82^

The XNA‐RT‐Cas12a system represents one of the most advanced implementations of this principle. In this design, an XNA blocking strand is positioned adjacent to the methylation site, inhibiting full‐length cDNA synthesis under normal conditions. When the RNA contains m6A, its weakened A‐U pairing and increased local flexibility allow partial displacement of the XNA or alter the interaction between RNA and reverse transcriptase, generating truncated or mismatch‐containing cDNA fragments. These products are subsequently converted into double‐stranded DNA through PCR, and the resulting dsDNA contains methylation‐dependent sequence or structural differences that activate Cas12a with high specificity (Figure 2a).^79^ This strategy enables direct discrimination of methylated and unmethylated RNA at single‐nucleotide resolution without requiring chemical derivatization or immunoprecipitation.

Beyond m6A, other RNA modifications similarly impart RT‐readable signatures. m1A, which disrupts Watson‐Crick hydrogen bonding and can introduce positive charge at N1, typically induces strong RT stops or truncated cDNA. m5C affects polymerase fidelity and readthrough efficiency, generating reproducible mutation or RT pausing patterns. Once transcribed into dsDNA, these modification‐dependent cDNA products can be readily analyzed using Cas12a or Cas12b, with collateral cleavage providing quantitative signal amplification.^83^

By leveraging the intrinsic sensitivity of reverse transcriptase to RNA chemical modifications, RT‐mediated CRISPR detection transforms structurally encoded epitranscriptomic signals into precise DNA‐level features. This approach enables highly specific, bisulfite‐free, and antibody‐free RNA methylation analysis while maintaining compatibility with low‐input RNA and standard Cas12 detection workflows, making it a versatile foundation for CRISPR‐based epitranscriptomic sensing.

#### 3.2.2 Cas13 direct sensing of methylation‐induced structural changes of m6A

Unlike RT‐mediated Cas12a assays, Cas13‐based strategies can detect RNA methylation without converting the RNA target into a DNA intermediate. A representative example is the CRISPRm6A assay, which exploits the methylation‐sensitive cleavage activity of MazF. MazF selectively cleaves unmethylated ACA‐containing RNA, whereas m6A modification at the corresponding site protects the RNA target from cleavage. The preserved methylated RNA subsequently activates Cas13a, whose collateral cleavage releases activators for Csm6 and initiates a tandem CRISPR amplification cascade (Figure 2b).^80^ This preamplification‐free workflow enables highly sensitive and single‐base‐resolution detection of site‐specific m6A.

m6A induces characteristic local structural rearrangements, often referred to as “m6A switches,” by destabilizing A‐U base pairing and promoting base flipping or partial helix melting. These alterations increase the flexibility of surrounding nucleotides and can transition structured motifs such as hairpins or internal loops into more open conformations.^84^ Cas13 activation efficiency varies markedly depending on RNA folding state, with even small differences in accessibility producing substantial changes in collateral cleavage output.^85^ Thus, when methylation perturbs local RNA architecture, Cas13 can distinguish modified from unmodified transcripts based on structure‐dependent hybridization efficiency rather than direct sequence differences.

Emerging studies on Cas13b further support this principle. m6A can reshape RNA–protein and RNA–RNA interactions by altering RNA structural ensembles, and these methylation‐induced conformational changes may also affect the formation of productive Cas13 crRNA‐target complexes.^86^ This suggests that Cas13 can function as a structure‐responsive biosensor, capable of detecting epitranscriptomic marks through differences in target accessibility, without requiring reverse transcription, chemical derivatization, or antibody enrichment.

Although still an evolving research direction, structure‐based Cas13 sensing offers the potential for direct, amplification‐free RNA methylation detection, particularly for modifications like m6A and pseudouridine that produce strong local conformational changes. By exploiting the intrinsic coupling between RNA folding energetics and Cas13 binding, this approach opens a promising pathway toward rapid and minimally processed epitranscriptomic diagnostics.

#### 3.2.3 Expanding beyond m6A

Although m6A is the most extensively studied RNA modification in CRISPR‐based assays, other epitranscriptomic marks, including m5C, m1A, and pseudouridine (Ψ), also induce biochemical effects that can be exploited by RT‐mediated or structure‐sensitive CRISPR platforms. Each modification perturbs RNA chemistry in distinct ways, producing characteristic signatures that can be leveraged to develop next‐generation CRISPR diagnostic systems.

m5C subtly modulates C‐G base pairing and influences local RNA secondary structure, often leading to measurable changes in reverse transcription fidelity or misincorporation rates. These RT‐derived mutation or pausing patterns can, in principle, be amplified and recognized by Cas12 nucleases, mirroring the strategy used for m6A. The broader distribution of m5C across tRNA, rRNA, and mRNA suggests that CRISPR‐compatible m5C assays may enable monitoring of diverse RNA classes beyond protein‐coding transcripts.^87^

Beyond m5C, CRISPR‐based sensing has also been extended to m1A. Because methylation at the N1 position of adenosine disrupts canonical Watson‐Crick base pairing, m1A within the crRNA‐target duplex suppresses productive Cas13a activation and reduces collateral cleavage activity. This differential Cas13a response enables direct discrimination of methylated and unmethylated RNA targets without reverse transcription or chemical conversion (Figure 2c).^81^ The strong perturbation of base pairing caused by m1A therefore provides a particularly favorable molecular signature for direct CRISPR‐based detection.

Ψ (pseudouridine) alters RNA folding by enhancing base stacking and modifying local rigidity.^88^ These structural perturbations are ideally suited to Cas13‐based sensing, as Cas13 activity is strongly influenced by target accessibility.^85^ A methylation‐ or isomerization‐induced shift in RNA conformation could modify crRNA:target hybridization efficiency, enabling structure‐dependent Cas13 activation analogous to m6A sensing.^89^

Despite their promise, CRISPR detection of these modifications remains underdeveloped. Key challenges include the modest and context‐dependent structural effects of m5C and Ψ, the need for high‐fidelity amplification of RT signatures, and limited understanding of how individual modifications alter Cas13 binding kinetics. Nonetheless, the distinct biochemical footprints of m5C, m1A, and Ψ provide rich opportunities for transcriptome‐wide epitranscriptomic profiling using CRISPR platforms. As probe engineering, RT‐based conversion chemistries, and structure‐aware Cas13 systems continue to evolve, CRISPR technologies are poised to expand from m6A toward a comprehensive suite of RNA modification sensors, enabling single‐nucleotide, isoform‐resolved, and potentially amplification‐free analysis of the full epitranscriptome.

## 4 TRANSLATIONAL CHALLENGES, EMERGING OPPORTUNITIES, AND FUTURE DIRECTIONS

### 4.1 Current stage of clinical and industrial translation

DNA methylation diagnostics have achieved clinical and commercial translation across several cancer indications. Representative assays, together with their biomarkers, biospecimens, manufacturers, detection technologies, and intended clinical applications, are summarized in Table 6. These products establish the clinical utility and translational feasibility of methylation biomarkers, although most depend on multistep sample processing, specialized instrumentation, and centralized laboratory infrastructure. CRISPR‐based detection may provide a complementary approach by combining programmable sequence recognition and rapid signal generation with compatibility with short nucleic acid targets and portable readout formats.^90^, ^91^

**TABLE 6:** Commercially available and clinically translated DNA methylation assays.

Test name	Biosample	Methylation biomarker	Manufacturer	Detection technology	Intended clinical use
ConfirmMDx™^92^	Prostate biopsy	GSTP1, APC, RASSF1	MdxHealth	Bisulfite +qMSP	Detection of occult prostate cancer
EpiCheck®^93^	Voided urine	Over 15 methylation markers	Nucleix	Enzyme + PCR	Surveillance of nonmuscle‐invasive bladder cancer (NMIBC)
UriFind®^94^	Voided urine	ONECUT2, VIM	AnchorDx	Bisulfite +qMSP	For auxiliary diagnosis of suspected urothelial cancer in patients undergoing initial diagnosis
therascreen®^95^	Breast tumor tissue	PITX2	Qiagen	Bisulfite +qMSP	Predict response to anthracycline‐based chemotherapy
Cologuard®^96^	Stool	NDRG4, BMP3 (+ KRAS mutation, occult hemoglobin)	Exact Sciences	Bisulfite +qMSP	Detection of colorectal cancer (CRC)
COLVERA™^97^	Plasma	IKZF1, BCAT1	Clinical Genomics	Bisulfite +qMSP	Detection of residual disease of CRC post‐surgical resection
ColoTect®^98^	Stool	SDC2, ADHFE1, PPP2R5C	Envelope Health Biotechnology	Bisulfite +qMSP	Detection of early CRC and precancerous lesions
Colon9®^99^	Plasma	SEPT9	Tellgen	Bisulfite +qMSP	Aid in the detection of CRC
ColonUSK®^100^	Plasma	SEPT9	USKbio	Bisulfite +qMSP	Aid in the early detection of CRC
ColonAiQ®^101^	Serum	Septin9, BCAT1, IKZF1, BCAN, VAV3	Singlera Health	Bisulfite +qMSP	Aid in the detection of CRC
CISCER®^102^	Cervical exfoliated cells	PAX1, JAM3	Beijing OriginPoly Bio‐Tec	Bisulfite +qMSP	Used for cervical cancer screening diversion
HCC Blood Test^103^	Plasma	SEPT9	Epigenomics	Bisulfite +qMSP	Detection of hepatocellular carcinoma
IvyGene®^104^	Blood, 40 mL	46 markers	Laboratory for Advanced Medicine	Gene sequencing	Detection of breast, colon, liver, and lung cancer
LungMe®	Bronchoalveolar lavage fluid (BALF)	SHOX2, RASSF1A	Tellgen	Bisulfite +qMSP	Aid in the detection of lung cancer
Si Bo Wei®	Plasma	RNF180, Septin9	BioChain	Bisulfite +qMSP	Detection of gastric cancer in high‐risk individuals
Ai Wei Ding®	Plasma	Reprimo, SDC2, TCF4	Beijing Excellen Medical Technology	Bisulfite +qMSP	Detection of gastric cancer

Abbreviation: qMSP, quantitative methylation‐specific real‐time PCR.

In contrast to established methylation assays, CRISPR‐based methylation detection remains largely at the laboratory proof‐of‐concept or early prototype stage. Reported platforms have demonstrated capabilities such as single‐site or single‐CpG discrimination, attomolar sensitivity when coupled with target amplification, and bisulfite‐free workflows that may reduce damage to low‐input or fragmented DNA. Nevertheless, most studies have been conducted using synthetic targets, purified nucleic acids, or limited clinical specimens, and none of the systems reviewed here has progressed to a formally approved clinical assay.^14^ Previous regulatory experience with CRISPR‐based infectious‐disease diagnostics provides a useful translational precedent, but equivalent analytical robustness and clinical performance have yet to be established for methylation biomarkers.

Overall, CRISPR methylation detection is transitioning from methodological proof of concept toward early translational development. Its progression will depend on validation in sufficiently large and representative clinical cohorts, direct benchmarking against established PCR‐ and sequencing‐based methylation assays, and demonstration of reproducibility across laboratories and sample types.^90^, ^91^

### 4.2 Technical and biological barriers

Clinically relevant methylated nucleic acids are often scarce, fragmented, and heterogeneous. Tumor‐derived cfDNA is enriched in short fragments, while disease‐associated methylation patterns may represent only a small fraction of total cfDNA.^105^, ^106^ Fragmentation can separate methylation sites from sequences required for amplification, enzymatic recognition, probe hybridization, or CRISPR targeting, particularly when single‐CpG detection requires a nearby PAM. Cell‐free RNA presents additional challenges because of its instability, secondary structure, and interactions with other biomolecules.^82^

Sequence and accessibility constraints also limit assay generalizability. Suitable PAMs or PFSs may be absent near the modification site, and nucleic acid structures or associated proteins can impede crRNA binding. Because most assays convert methylation into sequence‐, structure‐, or cleavage‐dependent differences, their performance depends on both the upstream discrimination step and subsequent Cas activation.^79^, ^80^, ^81^ PAM‐relaxed Cas variants may expand target coverage but can affect specificity, kinetics, and background activity.

Sample processing introduces further variability. Bisulfite treatment can degrade DNA or result in incomplete conversion, while methylation‐sensitive restriction enzymes may exhibit incomplete or sequence‐dependent digestion.^107^ RNA modification‐dependent reverse‐transcription signatures vary with the enzyme, reaction conditions, sequence context, structure, and modification stoichiometry.^108^ Amplification can improve sensitivity but may introduce bias and nonspecific products. In addition, collateral‐cleavage signals depend on crRNA efficiency, Cas kinetics, reaction conditions, and matrix inhibition, complicating quantification and multiplexing.^109^ Addressing these limitations will require streamlined sample preparation, appropriate controls, internal standards, and kinetic calibration.

### 4.3 Assay integration, manufacturing, and regulatory translation

Clinical translation requires CRISPR methylation assays to integrate sample preparation, methylation discrimination, signal generation, and readout into simple and reproducible workflows. Lateral‐flow formats may support rapid qualitative testing, whereas microfluidic, electrochemical, and cartridge‐based systems may be more suitable when quantitative measurements are required.^90^, ^91^ One‐pot or closed‐tube workflows could reduce sample loss, contamination, and operator variability. Portable integrated CRISPR systems have been demonstrated for infectious‐disease detection, although comparable performance remains to be established for methylation biomarkers in representative clinical specimens.^110^, ^111^

Scalable production of Cas proteins, crRNAs, reporters, methylation‐processing enzymes, and other reagents requires defined quality attributes, release criteria, traceability, and batch‐to‐batch consistency. Variations in reagent purity, integrity, activity, and labeling can affect reaction kinetics and background signals. Lyophilization may improve storage and distribution, but the performance of the complete assay must be validated throughout its intended shelf life.^105^ Standardization is further limited by the lack of commutable reference materials representing clinically relevant methylation fractions, fragment sizes, sequence contexts, and sample matrices. Interlaboratory evaluation of methylated cfDNA candidate reference materials provides a basis for harmonization,^106^ but comparable RNA standards remain less established.

Clinical validation should progress from reference materials and retrospective specimens to adequately powered, blinded, multicenter studies. Assays should be assessed for sensitivity, specificity, precision, robustness, interference, failure rate, and diagnostic thresholds and benchmarked against established methylation methods for quantitative agreement, turnaround time, workflow complexity, and cost. Regulatory planning should begin early because the intended use, specimen type, testing environment, multiplexing strategy, and data‐interpretation method determine the required evidence. Intellectual property, licensing, freedom to operate, reimbursement, manufacturing cost, and clinical workflow integration must also be considered.^107^ Rather than universally replacing PCR or sequencing, CRISPR may offer its greatest near‐term value in rapid or decentralized methylation testing where conventional laboratory infrastructure is limited.

### 4.4 Future Research and Development priorities

Near‐term development should focus on integrated, amplification‐free, and quantitatively reliable detection. Direct analysis of rare methylated molecules will require more active Cas effectors, optimized crRNAs and reporters, improved reaction kinetics, and efficient conversion of methylation‐dependent differences into Cas activation.^78^ PAM‐relaxed variants may broaden target coverage, while optimization of crRNA scaffolds, guide length, and reporter configuration may improve sensitivity and signal‐to‐background ratios.^108^ However, engineered systems must be evaluated using both methylated and unmethylated targets because increased activity or relaxed PAM recognition may compromise specificity.

Multiplexed DNA–RNA methylation profiling represents another important priority. Spatial compartmentalization, orthogonal Cas effectors, crRNA barcoding, and distinguishable reporters have enabled multiplexed CRISPR detection.^12^ Combining Cas12‐ and Cas13‐based systems could potentially support biomarker panels covering 5mC, 5hmC, m6A, and m1A, although this remains largely unvalidated. Progress will require compatible sample preparation, appropriate internal controls, suppression of collateral‐cleavage crosstalk, and calibration across biomarkers with different abundances and modification stoichiometries.

Artificial intelligence and structure‐guided modeling may accelerate guide, effector, and probe design. Deep learning has improved prediction of Cas12a guide activity,^109^ while AlphaFold 3 enables modeling of complexes containing proteins, nucleic acids, ions, and modified residues.^112^ Computational methods may also assist in interpreting kinetic signals and multidimensional biomarker panels, but future models should be trained on experimentally validated methylation‐assay datasets.

Beyond these near‐term priorities, CRISPR epigenetic analysis may eventually extend toward single‐cell, spatial, and long‐read profiling. Integration with droplet microfluidics, single‐cell sequencing, or spatial methylome transcriptome technologies could help resolve modification heterogeneity across cells and tissue regions.^113^ Cas9‐guided nanopore enrichment already enables targeted long‐read analysis of native DNA while preserving sequence, haplotype, and CpG methylation information.^114^ Live‐cell reporters may offer dynamic monitoring in the longer term, but modification‐sensitive recognition, low‐background reporting, and non‐perturbative detection remain substantial challenges. If these limitations can be addressed, CRISPR platforms may gradually expand beyond targeted endpoint detection toward multidimensional analysis of epigenetic heterogeneity and dynamics.

## 5 CONCLUSIONS

CRISPR‐based technologies provide a programmable approach for detecting DNA and RNA methylation by converting modification states into measurable signals through chemical conversion, methylation‐sensitive enzymes, reverse transcription, or modification‐dependent changes in target accessibility and Cas activity. Reported assays have demonstrated site‐specific discrimination, high analytical sensitivity, and potential compatibility with low‐input cell‐free nucleic acids and portable readout formats. However, most remain at the proof‐of‐concept stage and have been evaluated primarily using synthetic targets, purified nucleic acids, or limited clinical specimens. Reliable quantification of methylation fractions also remains challenging because signals are influenced by upstream processing or amplification efficiency, Cas reaction kinetics, and sample‐matrix effects.

The evidence base is currently stronger for DNA methylation detection than for RNA methylation analysis, which has been demonstrated in fewer studies and often relies on indirect signal‐conversion steps. Further progress will require standardized workflows, representative reference materials, quantitative calibration, and validation in sufficiently large clinical cohorts. In the near term, CRISPR platforms are therefore more likely to complement established PCR‐ and sequencing‐based methods, particularly for rapid, targeted, or decentralized testing, than to replace them. Their longer‐term clinical value will depend on translating analytical sensitivity into reproducible, scalable, and clinically informative diagnostic workflows.

## AUTHOR CONTRIBUTIONS

**Wenjie Chen:** Writing – review and editing. **Tingxiu Xiang:** Supervision; writing – review and editing. **Biyao Yang:** Investigation; writing – review and editing. **Kaixin Chen:** Investigation; writing – original draft. **Fei Deng:** Conceptualization; supervision; writing – review and editing. **Rui Sang:** Writing – review and editing.

## CONFLICT OF INTEREST STATEMENT

The authors declare no conflicts of interest.

## Data Availability

The data that support the findings of this study are available from the corresponding author upon reasonable request.
